# Predictors of long-term mortality after intertrochanteric fractures surgery: a 3-year retrospective study

**DOI:** 10.1186/s12891-022-05442-2

**Published:** 2022-05-19

**Authors:** Yao Lu, Qiang Huang, Yibo Xu, Cheng Ren, Liang Sun, Wenchao Dong, Ming Li, Hanzhong Xue, Zhong Li, Kun Zhang, Teng Ma, Qian Wang

**Affiliations:** 1 Department of Orthopaedic Surgery, Honghui Hospital, Xi’an Jiaotong University, Xi’an, 710054 Shaan’xi Province China; 2 Shaanxi University of Chinese Medicine, Xian yang, 710000 Shaanxi China

**Keywords:** Intertrochanteric fractures, Hemoglobin, TLC, Mortality

## Abstract

**Introduction:**

Intertrochanteric fractures are associated with high mortality rates; however, long-term data on survival and predictors remain scarce. Therefore, this study investigated risk factors associated with 3-year mortality in elderly patients with intertrochanteric fractures.

**Methods:**

In a retrospective study, 156 elderly patients with intertrochanteric fractures who underwent surgery between January 2017 to January 2018 at our center were included. Association-affecting variables, such as gender, age, time from injury to surgery, hemoglobin (Hb), total lymphocyte count (TLC), albumin, malnutrition, and co-morbidities, were recorded and analyzed. Afterward, logistic regression was used to analyze the significant variables and find independent predictors for 3-year mortality.

**Results:**

A total of 156 patients were followed up for 3 years. The 1-year, 2-year, and 3-year postoperative cumulative mortality rates were 9.6% (15/156), 16.7% (26/156), and 24.4% (38/156), respectively. Simple analyses found that age, Hb, albumin, and malnutrition were associated with 3-year mortality (*p* < 0.05). Multivariable analysis confirmed that advanced age (*p* < 0.001) and low albumin (*p* = 0.014) were independent risk factors for 3-year mortality.

**Conclusion:**

Low serum albumin and advanced age were independent risk factors for long-term mortality in elderly patients with intertrochanteric fractures.

**Supplementary Information:**

The online version contains supplementary material available at 10.1186/s12891-022-05442-2.

## Introduction

With rapid social development and an aging population, the incidence and prevalence of hip fractures are rapidly increasing, especially in developing countries. It is estimated that by 2050, in China, the number of elderly people aged over 60 will exceed 450 million and that the elderly population in China shall account for nearly 20% of the global elderly population [1, 2]. Hip fracture is a common cause of disability and death in the elderly and place a tremendous socioeconomic strain on society. Therefore, early investigation and management of the risk factors for poor prognosis after hip fractures are crucial.

Several studies reported that multiple factors, such as age, gender, blood albumin, hemoglobin (Hb), BNP, glucose, creatinine, arterial blood pH, partial pressure of oxygen, and neutrophil-to-lymphocyte ratio are associated with high mortality after hip fractures [3, 4]. Among the risk factors for loss of function after hip fractures, malnutrition is an area of great interest, mainly because it is modifiable [5]. Malnutrition predisposes one to hip fractures, is a common precipitating factor for fractures, and has a predictive value for mortality in the first year after fracture surgery [5]. Albumin and total lymphocyte count (TLC) are nutritional markers that can be used to define malnutrition when albumin is < 35 g/L and TLC is < 1.5 × 10^9^ cells/L [6]. Malnutrition has an impact on surgical incision healing, and serum albumin, TLC, hemoglobin, and age are mentioned in several studies as independent predictors of postoperative mortality [5, 7].

However, most studies have focused on hip fractures. Hip fractures include femoral intertrochanteric fractures and femoral neck fractures. There are differences between these two fractures (patient characteristics, surgical treatment, and outcome). According to a systematic analysis, the 1-year mortality rate after femoral neck fractures in China is 9.83%, while that of intertrochanteric fracture is 17.47% [8].

Therefore, there is a need for a predictive risk assessment for a single fracture. This study aimed to investigate risk factors associated with 3-year mortality in elderly patients with intertrochanteric fractures after intramedullary fixation.

## Materials and methods

### Patients

This retrospective study included all elderly patients with intertrochanteric fractures at our center (level-1 trauma center) between January 2017 to January 2018. The inclusion criteria were as follows: (1) patients with intertrochanteric fractures, (2) age ≥ 65 years, (3) patients who underwent internal fixation by proximal femoral nail antirotation (PFNA), and (4) postoperative follow-up ≥ 3 years. The exclusion criteria were the following: (1) pathological fractures or open fractures; (2) patients with multiple fractures; (3) patients who had preoperative infections, severe immunodeficiency, liver disease, or blood disorders; (4) refused follow-up; and (5) patients with incomplete clinical data before and/or after surgery. This study was reviewed and approved by the Ethics Committee of Honghui Hospital, Xi’an Jiaotong University. All participating patients provided signed informed consent.

## Methods

**Fig. 1:**
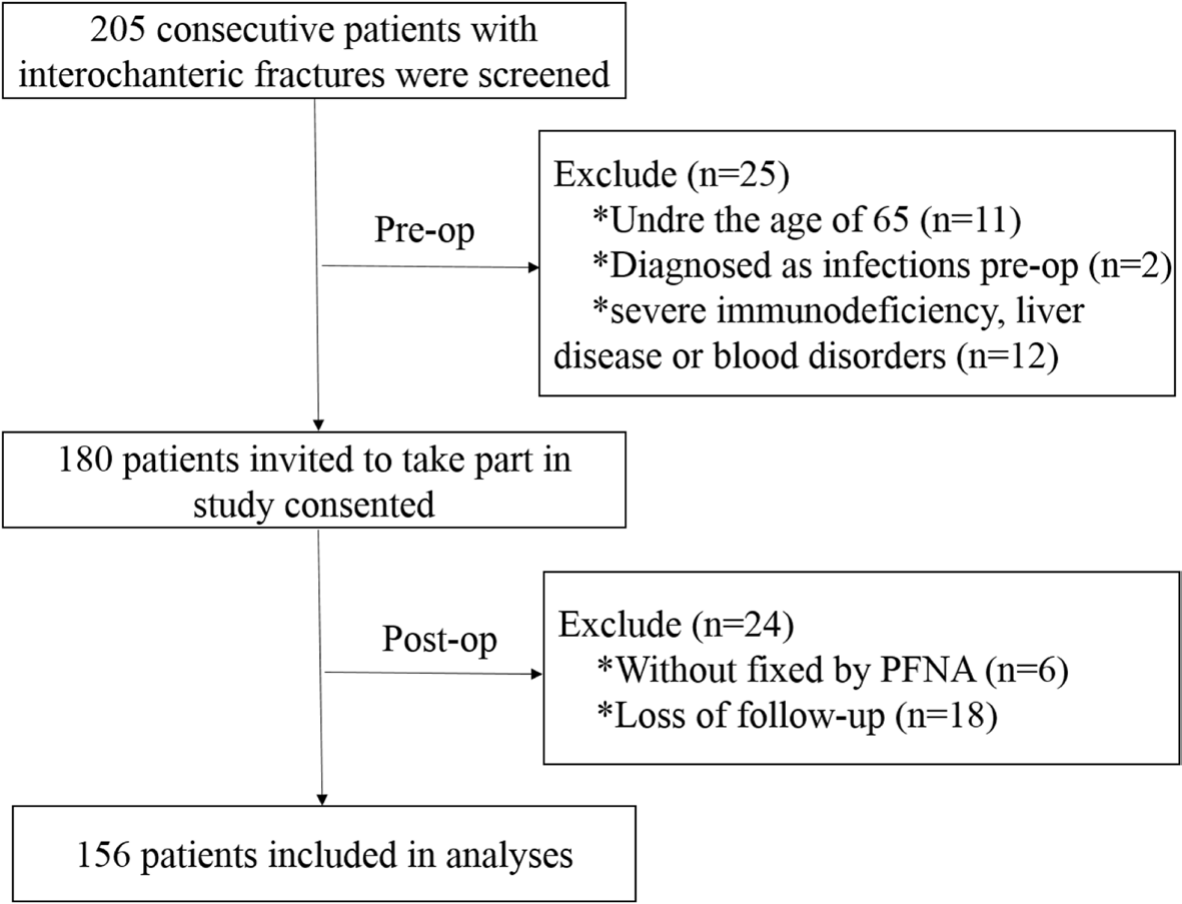
Flow diagram of patient selection

The clinical data of patients with intertrochanteric fractures between January 2017 and January 2018 were collected through the hospital electronic medical record system. The patients’ characteristics were extracted as follows: gender, age, time from injury to surgery, Hb level at admission (g/L), TLC level at admission (cells/L), Albumin (g/L), malnutrition, comorbidities from patients' histories (including hypertension, diabetes, heart disease, cerebral apoplexy), and deep vein thrombosis [(DVT) duration of hospital stay] in the lower limbs. Patients were routinely given rehydration (Sodium Lactate Ringer 500 ml) at admission, and Hb sample collection on the second day of admission Using the inclusion and exclusion criteria, 49 patients were excluded. A total of 156 patients meeting the criteria were assessed after complete enumeration (Fig. 1). Afterward, 1-year, 2-year, and 3-year mortality were recorded.

### Statistical analysis

Statistical analyses were computed using SPSS 22. The Shapiro–Wilk test was used to determine whether the continuous variables were normally distributed. Data satisfying normality were reported as means and standard deviations. A non-parametric test or Student’s t-test was used to compare the differences between two groups, and the chi-square or Fisher test was used for the analysis of categorical data. Univariate logistic regression models were used to validate risk factors for mortality. When *P* < 0.1, multifactorial logistic regression models were used to validate independent risk factors for mortality. *P*-values < 0.05 indicated a statistically significant difference.

## Results

**Fig. 2:**
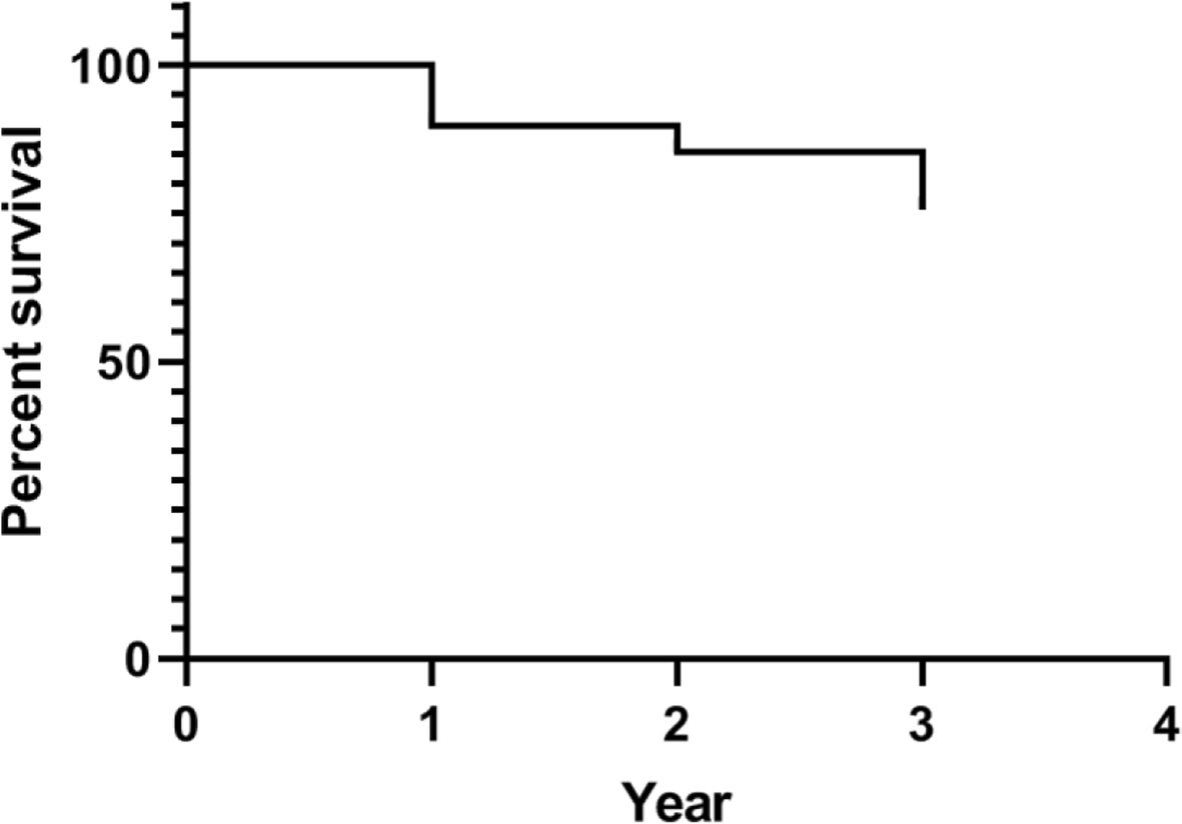
Kaplan–Meier curve showing 3-year survival

A total of 205 consecutive patients with intertrochanteric fractures were screened between January 2017 and January 2018, and their eligibility for participation was assessed in this study. A total of 49 patients were excluded according to the exclusion criteria, including 11 patients under 65 years of age, 2 patients with preoperative infection, 12 patients with severe immunodeficiency or blood disorders, 18 patients lost to follow-up, and 6 patients who received non-PFNA fixation. Finally, a total of 156 patients with intertrochanteric fractures were enrolled in the present study (Fig. 1), with 9.6% (*n* = 15), 7.1% (*n* = 11), and 7.7% (*n* = 12) of patients dying in the first, second, and third years, respectively. Therefore, the 1-year, 2-year, and 3-year postoperative cumulative mortality rates were 9.6% (15/156), 16.7% (26/156), and 24.4% (38/156), respectively (Fig. 2).

There were 55 men and 101 women, with a mean age of 81 years (range, 65–96 years). The average Hb, TLC, and albumin were 105.3 ± 16.9 g/L, 1.17 ± 0.51 × 10^9^ cells/L, and 36.6 ± 3.9 g/L, respectively. There were 45 (29%) patients who underwent surgery within 48 h of injury, and 111 (71%) patients experienced a delay in surgery of > 48 h after injury. There were 63, 27, 93, 41, and 75 cases of hypertension, diabetes, heart disease, cerebral apoplexy, and DVT, respectively. There were 41 patients with malnutrition. The patients’ baseline data are provided in Table 1.

**Table 1:** General data

Variables	*n* = 156	(Mean ± SD)%
Gender		
Male	55	35%
Female	101	65%
Age		
Total		81(65–96)
Lived > 3 years	118	79(65–94)
Died within 3 years	38	85(67–96)
Time from injury to surgery		
< 48 h	45	29%
≥ 48 h	111	71%
Hb level at admission (g/L)		
Total		105.3 ± 16.9
Lived > 3 years	118	107.1 ± 17.1
Died within 3 years	38	99.6 ± 15.1
TLC level at admission (× 10^9^ cells/L)		
Total		1.17 ± 0.51
Lived > 3 years	118	1.23 ± 0.54
Died within 3 years	38	1.01 ± 0.35
Albumin (g/L)		
Total		36.6 ± 3.9
Lived > 3 years		37.3 ± 3.7
Died within 3 years		34.5 ± 3.5
Malnutrition		
Yes	41	26%
No	115	74%
Hypertension	63	40%
Diabetes	27	17%
Heart disease	93	59%
Cerebral apoplexy	41	26%
DVT	75	48%

*TLC* total lymphocyte count, *DVT* deep vein thrombosi

Univariate analysis showed that there was no statistical difference in gender, delay in surgery, TLC level at admission, DVT, medical history (diabetes, hypertension, heart disease, and cerebral apoplexy), and the number of co-morbidities (*p* > 0.05) between the living and dead groups, whereas there were significant differences in age, Hb, albumin, and malnutrition (*p* < 0.05; see Table 2). The levels of albumin, TLC, and Hb were 34.5 ± 3.5 g/L, 1.01 ± 0.35 × 10^9^ cells/L, and 99.6 ± 15.1 g/L, respectively, in the dead group, and 37.3 ± 3.7 g/L, 1.23 ± 0.54 × 10^9^ cells/L, and 107.1 ± 17.1 g/L, respectively, in the living group. There were statistical differences between the two groups in these three variables (*p* < 0.05); see Table 3.

**Table 2:** Simple analysis of predictors of mortality

Variables	*n* = 156	Living	Dead	P
		*n* = 118	*n* = 38	
Gender				0.349
Male	55	44	11	
Female	101	74	27	
Age				< 0.001
65–74	35	33	2	
75–84	76	64	12	
≥ 85	45	21	24	
Time from injury to surgery				0.987
< 48 h	45	34	11	
≥ 48 h	111	84	27	
Hb level at admission (g/L)				0.003
> 120	28	26	3	
100–120	72	59	12	
80–100	44	25	19	
< 80	12	8	4	
TLC level (× 10^9^ cells/L)				0.097
> 1.5	31	27	4	
≤ 1.5	125	91	34	
Albumin (g/L)				< 0.001
≥ 35	105	89	16	
< 35	51	29	22	
Malnutrition				< 0.001
Yes	41	20	21	
No	115	98	17	
Hypertension				0.609
Yes		49	14	
No		69	24	
Diabetes				0.204
Yes		23	4	
No		95	34	
Heart disease				0.372
Yes		68	25	
No		50	13	
Cerebral apoplexy				0.668
Yes		30	11	
No		88	27	
DVT				0.352
Yes		52	23	
No		66	21	
Co-morbidities				0.904
< 2		67	22	
≥ 2		51	16

**Table 3:** Comparison of albumin, TLC, and Hb levels between two groups

Group	n	Albumin	TLC	Hb
Living group	118	37.3 ± 3.7	1.23 ± 0.54	107.1 ± 17.1
Dead group	38	34.5 ± 3.5	1.01 ± 0.35	99.6 ± 15.1
t		4.109	2.359	2.378
P		< 0.001	0.02	0.019

**Fig. 3:**
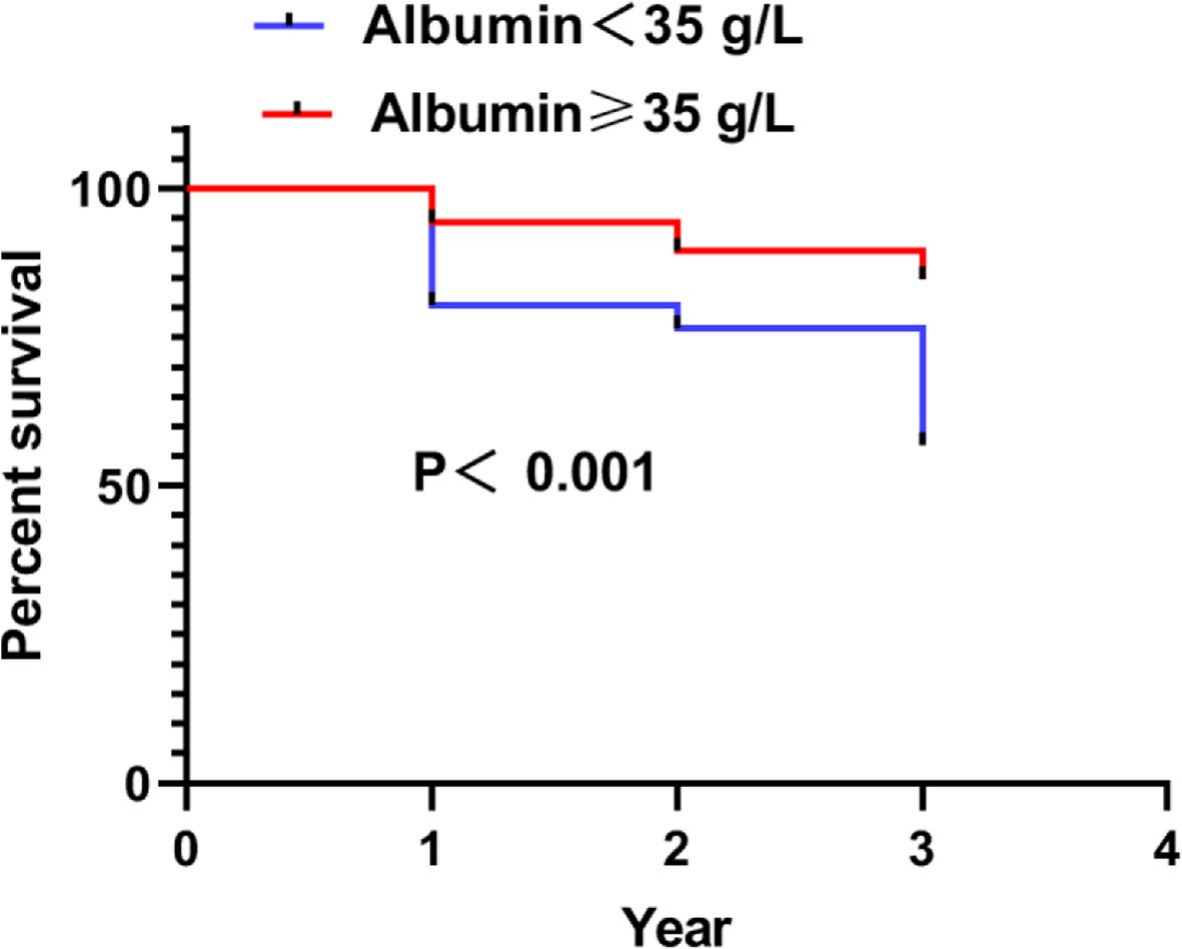
Kaplan–Meier survival curves showing that those with high albumin had a longer survival time than those with low albumin

Multivariate logistic regression analysis showed a significant difference in 3-year mortality in age and albumin (*p* < 0.05). Older patients had an OR of 5.169 (95% CI: 3.199–8.351, *p* < 0.001) compared to younger patients. Patients with a low albumin level at admission had an OR of 5.093 (95% CI: 1.397–18.567, *p* = 0.014) compared to patients with a high albumin level. There were no significant differences in Hb, TLC, and malnutrition (*p* < 0.05). However, patients with a low TLC level at admission had an OR of 2.28 (95% CI: 0.897–5.799, *p* = 0.084) compared to patients with a high TLC level; patients with a low Hb level at admission had an OR of 1.367 (95% CI: 0.963–1.939, *p* = 0.080) compared to patients with a high Hb level. The above results revealed that older age and a low albumin level were independent predictors of mortality within three years (see Table 4). Finally, according to Kaplan–Meier survival curves, the survival time of patients in the albumin ≥ 35 g/L group was longer than those in the albumin < 35 g/L group (X^2^ = 15.169, *p* < 0.001; see Fig. 3).

**Table 4:** Multivariate analysis of significant predictors

Indicators	Age	Hb	Albumin	TLC	Malnutrition
B	1.643	0.312	1.628	0.824	-0.435
SE	0.245	0.178	0.660	0.476	0.698
Wald	45.047	3.066	6.082	2.995	0.389
P	< 0.001	0.080	0.014	0.084	0.533
OR	5.169	1.367	5.093	2.28	0.647
95% CI					
Lower limit	3.199	0.963	1.397	0.897	0.165
Upper limit	8.351	1.939	18.567	5.799	2.542

## Discussion

With the aging of the global population, intertrochanteric fractures, which have a high postoperative mortality rate, are a growing public health problem in many parts of the world, especially in developing countries [9]. A meta-analysis investigated 75 studies with 64,316 patients and found that 1-year and 2-year mortality rates were 24.5% and 34.5%, respectively [10]. Tiihonen et al. [11] reported that 1-year mortality rates were 22.2% in elderly patients with hip fractures. Although the 1-year, 2-year, and 3-year postoperative cumulative mortality rates (9.6%, 16.7%, and 24.4%, respectively) in this study were lower than above, 24.4% at the 3-year follow-up is still nearly one in four patients. The mortality rates were lower in our study than in previous studies. On the one hand, people in nursing home had the higher mortality rates in Europe and America [12, 13]. Most older Chinese people live with their children because of their traditional culture. They can get much better care. On the other hand, the economy has an important impact on the prognosis of hip fractures. Jacobs et al. [12] reported that patients from economically disadvantaged areas had an increase the mortality rate. With the rapid economic development in China over the past two decades, the mortality of diseases had a decrease [14]. Therefore, the postoperative mortality of patients with intertrochanteric fractures must be observed. Patients with intertrochanteric fractures often have more chronic diseases, such as diabetes, hypertension, coronary artery disease, and chronic bronchitis [9]. Older patients have a poorer appetite; therefore, patients with intertrochanteric fractures have a higher rate of malnutrition compared with the general population [6]. Malnutrition is also associated with muscle atrophy due to trauma or surgery, postoperative complications, and prolonged bed rest. Some studies have shown that albumin, hemoglobin, TLC, age, and sex are associated with mortality in patients with hip fractures [5, 6, 8]. However, there is a lack of predictors of postoperative mortality for intertrochanteric fractures. The results of this study showed that albumin and age were independent risk factors for mortality in elderly patients with intertrochanteric fractures. Further, other risk factors include Hb and malnutrition.

The impact of age on the prognosis of patients with intertrochanteric fractures remains controversial. Guo et al. [9] investigated 3560 patients with intertrochanteric fractures and showed that elderly patients had more perioperative complications and higher 1-year mortality than younger patients with intertrochanteric fractures after PFNA. However, after propensity score matching, they confirmed that age does not predict worse mortality. With increasing age, decreased organism function and hematopoietic function can lead to lower nutritional intake in patients, further increasing the risk of postoperative mortality. Rui and colleagues [15] investigated 135 patients with intertrochanteric fractures with PFNA and demonstrated that age was an independent risk factor for postoperative mortality. Similarly, the present study showed that elderly patients (with age ≥ 85 years) had a 5.169-fold higher risk of death than younger patients (with age < 85 years). Therefore, orthopedic surgeons must pay attention to the perioperative management of elderly patients with intertrochanteric fractures.

This study demonstrated that malnutrition is common among intertrochanteric fracture patients. Most studies used preoperative albumin levels as an indicator of nutritional status. In general, they found that a lower level of albumin at admission is associated with postoperative outcomes in elderly patients with intertrochanteric fractures [16, 17]*.* TLC is another important indicator of the immune status and nutritional status of an individual. For hospitalized patients, a decrease in TLC can lead to immune dysfunction or abnormalities, resulting in a higher probability of complications and an increased probability of postoperative infections and death [5, 18]. Daly et al. [5] found that serum albumin and TLC affected postoperative mortality, but many scholars do not recognize this effect [19, 20]. The results of this study showed that albumin was significantly associated with 3-year postoperative survival, while TLC did not influence 3-year postoperative survival. In other words, protein energy malnutrition is associated with higher mortality. Given the urgency of intertrochanteric fracture surgery, it is clinically difficult to correct nutritional deficiencies immediately; however, most of the literature suggests that nutrition remains a modifiable postoperative risk factor [20]. This suggests that management strategies should be used to optimize health care and to implement nutritional supplementation strategies.

Hb is considered a predictive factor of postoperative mortality in hip fractures. Kovar et al. [19] investigated a total of 3595 patients and suggested that Hb level at the time of admission is a useful and cost-effective parameter for predicting mortality in elderly hip fracture patients. In the present study, univariate analysis showed that a low Hb level at the time of admission was associated with postoperative mortality. However, after multivariate analysis, Hb level was not significantly associated with long-term mortality after intertrochanteric fractures.

The timing of surgery is widely considered to be associated with high mortality rate [21]. Therefore, current guidelines in developed countries recommend surgery within 48 h of injury [22–25]. However, many studies demonstrated that delay in surgery is not associated with high mortality [10, 26]. Ravi [26] divided patients (age ≥ 50 years) with intertrochanteric fractures into three groups, > 48 h vs ≤ 48 h, > 5 days vs ≤ 5 days, and > 7 days vs ≤ 7 days, according to the time from fracture to surgery. The authors found that there was no correlation between time to surgery and postoperative mortality. Similarly, Cher et al. [27] reported that delay of surgery (≥ 48 h) did not have an impact on mortality at 90 days follow-up but was a risk factor for long-term mortality up to 2 years. After adjusting for other factors, delay of surgery was not associated with postoperative mortality. In China, due to the lack of a protocol-based multidisciplinary model, these patients cannot undergo surgery within 48 h. In this study, only 29% (45/156) of patients were operated on within 48 h. Delay of surgery did not impact long-term mortality at the 3-year follow-up.

Most studies reported that having two or more comorbidities was a risk factor associated with high mortality [26, 27]. A meta-analysis, including 75 studies involving 64,316 patients, indicated that multiple comorbidities were predictors for 1-year mortality in elderly patients with hip fractures [10]. Cher and colleagues [27, 28] reported that the Deyo-Charlson Comorbidity Index analyzes risk for mortality after hip fracture surgery at 2-year follow-up. This study demonstrated that co-morbidity was not a predictor of mortality. This may be related to insufficient sample size and postoperative care.

There are some limitations to this study. First, it was a retrospective single-center study with a small sample size. Not all of the univariately tested parameter were included into logistic regression model. A large-scale prospective study is required to predict risk factors of mortality for intertrochanteric fractures. Second, this study did not obtain a specific time of death (such as the number of months after injury) for patients with intertrochanteric fractures; this variable may affect the results of the multivariate COX regression survival analysis. Lastly, risk factors that we focus on are at admission, and Hb was analyzed after fluid supplementation. Parameters of the in-hospital course [ASA scores (American Society of Anesthesiologists), consecutive Hb, TLC-parameters, complications] should been included. And stratification of the patients' functional baseline (Mobility, Activities of daily living) should been stratified.

## Conclusion

The current results showed that serum albumin level and age were excellent prognostic indicators of postoperative 3-year mortality in elderly patients with intertrochanteric fractures.

## Supplementary Information

**Additional file 1:** 

## Data Availability

All data generated or analysed during this study are included in this published article [and its supplementary information files].
